# Predictors and outcomes associated with the growth curves of self-efficacy beliefs in regard to anger and sadness regulation during adolescence: a longitudinal cross-cultural study

**DOI:** 10.3389/fpsyg.2023.1010358

**Published:** 2023-04-17

**Authors:** Laura Di Giunta, Carolina Lunetti, Jennifer E. Lansford, Nancy Eisenberg, Concetta Pastorelli, Dario Bacchini, Liliana Maria Uribe Tirado, Anne-Marie R. Iselin, Emanuele Basili, Giulia Gliozzo, Ainzara Favini, Flavia Cirimele, Chiara Remondi

**Affiliations:** ^1^Department of Psychology, Sapienza University of Rome, Rome, Italy; ^2^Center for Child and Family Policy, Duke University, Durham, NC, United States; ^3^Department of Psychology, Arizona State University, Tempe, AZ, United States; ^4^Department of Human Science, University of Naples Federico II, Naples, Italy; ^5^Department of Psychology, Universidad de San Buenaventura Medellín, Medellín, Colombia; ^6^Department of Psychology, Elon University, Elon, NC, United States; ^7^Santa Lucia Foundation, Rome, Italy

**Keywords:** self-efficacy beliefs, parenting, adolescence, anger regulation, sadness regulation, growth curve

## Abstract

**Introduction:**

This longitudinal study examined unique and joint effects of parenting and negative emotionality in predicting the growth curves of adolescents’ self-efficacy beliefs about regulating two discrete negative emotions (anger and sadness) and the association of these growth curves with later maladjustment (i.e., internalizing and externalizing problems).

**Methods:**

Participants were 285 children (T1: *M*_*age*_ = 10.57, *SD* = 0.68; 53.3% girls) and their parents (mothers *N* = 286; fathers *N* = 276) from Colombia and Italy. Parental warmth, harsh parenting, and internalizing and externalizing problems were measured in late childhood at T1, whereas early adolescents’ anger and sadness were measured at T2 (T2: *M*_*age*_ = 12.10, *SD* = 1.09). Adolescent self-efficacy beliefs about anger and sadness regulation were measured at five time-points from T2 to T6 (T6: *M*_*age*_ = 18.45, *SD* = 0.71), and internalizing and externalizing problems were measured again at T6.

**Results:**

Multi-group latent growth curve models (with country as the grouping variable) demonstrated that in both countries there was on average a linear increase in self-efficacy about anger regulation and no change or variation in self-efficacy about sadness regulation. In both countries, for self-efficacy about anger regulation (a) T1 harsh parenting and T1 externalizing problems were negatively associated with the intercept, (b) T2 anger was negatively associated with the slope, and (c) the intercept and the slope were associated with lower T6 internalizing and externalizing problems, controlling for T1 problems. For self-efficacy about sadness regulation, (a) T1 internalizing problems were negatively associated with the intercept only in Italy, (b) T2 sadness was negatively associated with the intercept only in Colombia, and (c) the intercept negatively predicted T6 internalizing problems.

**Discussion:**

This study advances knowledge of the normative development of self-efficacy beliefs about anger and sadness regulation during adolescence across two countries, highlighting the predictive value of pre-existing family and individual characteristics on this development and prediction by the development of self-efficacy beliefs on later adjustment.

## Introduction

In line with differential susceptibility theory (Belsky and Pluess, 2009) and with the bioecological theory of development (Bronfenbrenner and Morris, 2006), the present study examines how multiple systems, including individual characteristics and contextual/family characteristics, contribute to predicting human development. In particular, the focus of this study was threefold: first, testing whether growth in a specific feature of personality, namely, self-efficacy beliefs in the domains of anger and sadness regulation, is predictive of internalizing and externalizing symptoms during adolescence; second, testing whether parenting and children’s anger and sadness are predictive of growth in either form of adolescents’ self-efficacy; and third, testing the aforementioned hypotheses across two countries, namely, Colombia and Italy.

The developmental transition to adolescence is associated with several challenges in biological, cognitive, emotional, and social systems (Steinberg and Morris, 2001). How adolescents face those challenges influences their psychological adjustment and long-term outcomes. Furthermore, adolescence is a critical stage of human development, characterized by an overall decrease in positive affect and a significant increase in the experience of negative emotion, with consequences for youths’ maladjustment (Griffith et al., 2021). In this context, it is useful to identify developmental trajectories of factors that could promote adolescents’ psycho-social adjustment. Among those factors, empirical findings (e.g., Bi et al., 2022) support the importance of self-efficacy, defined as the “*awareness of being able to dominate specific activities, situations or aspects of one’s psychological or social functioning*” (Bandura, 1997; p. 8). In the domain of emotion regulation, self-efficacy beliefs regarding individuals’ dealing with negative emotions are associated in similar ways across cultures, cross-sectionally and longitudinally, with low maladjustment and high adaptive behaviors in adolescence and adulthood (e.g., Bandura et al., 2003; Di Giunta et al., 2017, 2018).

Commonly, developmental research identifies individuals’ adjustment and maladjustment by examining two major macro-areas: externalizing and internalizing problems (e.g., Achenbach, 1991; Graber, 2004). Internalizing symptoms include symptoms related to anxiety and depression and focuses on individuals’ internal expression of distress; externalizing symptoms are directed outwardly and includes symptoms related to aggression and delinquency (e.g., Achenbach and Rescorla, 2006). Especially during adolescence, a focus on adjustment in terms of internalizing and externalizing symptoms can help to clarify the development of common psychiatric disorders such as anxiety, depressive, and conduct disorders, and to intervene in an effective and targeted way (e.g., Achenbach and Rescorla, 2006; Cosgrove et al., 2011; Rothenberg et al., 2022).

Previous studies have theoretically and statistically supported the existence of specific sub-dimensions of self-efficacy about the regulation of negative emotions, namely, self-efficacy about anger and sadness regulation. We focused on anger and sadness because of the strong association between their dysregulation and both externalizing (e.g., Zeman et al., 2002; Morris et al., 2011) and internalizing symptoms (Eisenberg and Eggum, 2009; Sanders and Mazzucchelli, 2013; Folk et al., 2014). In addition, self-efficacy about anger regulation has been uniquely and negatively associated with irritability, hostile rumination, and externalizing problems, whereas self-efficacy about sadness regulation has been uniquely and negatively related to sadness and depressive rumination, and internalizing problems (e.g., Caprara et al., 2008; Di Giunta et al., 2017).

The present study goes beyond the prior research because this is the first study examining: (1) the development of self-efficacy regarding anger and sadness regulation during adolescence (i.e., their developmental growth curves); (2) the predictive role of individual factors, such as temperamental anger and sadness, environmental factors, such as parenting, and the interaction between those two levels on the growth of self-efficacy about anger and sadness regulation; (3) the association between the growth of self-efficacy about anger and sadness regulation and adolescents’ later maladjustment (internalizing and externalizing problems). Moreover, to maximize the external validity of the results, the aforementioned aims were examined in two countries, Colombia and Italy.

Overall, the vast majority of the aforementioned associations have been examined with a correlational (albeit sometimes longitudinal) perspective—focusing on rank-order stability rather than mean-level change in self-efficacy (e.g., Roberts et al., 2006). To our knowledge, this is the first study examining the development of self-efficacy about the regulation of two discrete negative emotions and examining the associations of both hypothesized predictors and outcomes with the development of these specific self-efficacy beliefs across adolescence.

Furthermore, in accordance with the socio-cognitive theory (Bandura and National Institute of Mental Health [NIMH], 1986; Bandura, 2001), self-efficacy development is affected by the triadic mutual influences among internal personal factors (cognitive, affective, and biological), behavioral factors, and environmental factors (Bandura, 1997, 2001). In this theory, socio-economic status is an aspect of the environment that plays an important role in the development of self-efficacy. Indeed, individuals who experience greater economic pressure and limited financial resources might have lower self-efficacy than individuals who live in privileged circumstances and have more resources to support the beliefs in their successes and a sense of self-efficacy. For this reason, we included parents’ level of education as a covariate, because it is frequently used as an indicator of family socio-economic status.

## Longitudinal development of self-efficacy beliefs about emotion regulation

Empirical work on the developmental trajectories of self-efficacy about negative emotions is sparse. Caprara et al. (2013) examined the joint growth curve models of emotional stability (low neuroticism), self-efficacy beliefs in dealing with overall negative emotions, and self-efficacy in expressing positive emotions in the transition from 15 to 21 years old. Of interest to the current study, they found no linear growth in the best-fitting model of self-efficacy about dealing with overall negative emotions (i.e., averaging anger and sadness).

## Parenting and self-efficacy beliefs in emotion regulation

Parental behaviors significantly affect adolescents’ self-regulation and psycho-emotional development (e.g., Steinberg et al., 1994; Eisenberg et al., 1998). A wide range of behaviors related to parental warmth and authoritative parenting have been associated with adolescents’ positive outcomes, whereas authoritarian and harsh parenting have been associated with negative outcomes (Di Giunta et al., 2020; Lunetti et al., 2022).

In the present study, we focused on two parenting constructs in relation to adolescents’ self-efficacy beliefs about dealing with negative emotions, namely, parental warmth (as an indicator of positive parenting) and harsh parenting (as an indicator of negative parenting). Parental warmth is considered a supportive parental strategy characterized by expressing sensitivity toward children’s needs and being supportive, communicative, and responsive (Rohner, 2005). Harsh parenting is considered a non-supportive strategy in which parents are emotionally dysregulated and aggressive (e.g., Scaramella et al., 2008). Harsh parenting includes behaviors such as yelling, threatening, and intimidating verbally and physically (Hoskins, 2014), all behaviors that have negative consequences for youths’ development (e.g., Burnette et al., 2012; Hinnant et al., 2015; Lansford et al., 2018; Rothenberg et al., 2022).

Moreover, parents’ control (i.e., parents’ attempts to control adolescents’ thoughts and feelings) and rejection (including high levels of hostility, undifferentiated rejection, neglect, and low levels of warmth), when their offspring were 15 years old, were differentially associated with self-efficacy about anger versus sadness regulation at age 16 (Di Giunta et al., 2022). Specifically, parental control but not rejection was significantly associated with lower self-efficacy regarding anger regulation, and parental rejection but not control was significantly associated with lower self-efficacy in sadness regulation. That study supports the role of parenting in adolescents’ self-efficacy in the domain of emotion regulation and highlights the importance of considering the role of discrete emotions when examining the association between parenting and self-efficacy about emotion regulation.

In addition, Niditch and Varela (2012) demonstrated that youths aged 12 to 18 years old, who perceived their parents as more authoritarian, reported lower self-efficacy beliefs about their ability to cope with negative emotions, compared to those who perceived their parents as less authoritarian. Furthermore, recent studies have demonstrated the moderator and mediator effects of self-efficacy beliefs about managing negative emotions in the associations between parenting and children’s outcomes. For example, in a sample of Chinese junior high school students, students’ self-efficacy about managing negative emotions mediated the relation between parental autonomy granting (as an indicator of positive parenting) and their life satisfaction (Bi et al., 2022). In a sample of senior primary school students, Liu et al. (2022) found that for those children with high self-efficacy in managing negative emotions, prediction by harsh parenting of their aggressive behaviors was weaker than in those children with low self-efficacy.

### Self-efficacy beliefs in emotion regulation and adjustment

Our desire to examine the reciprocal associations between self-efficacy in regard to anger and sadness regulation and maladjustment is consistent with two theoretical perspectives: (1) a broad social-cognitive theoretical framework (Caprara and Cervone, 2000; Bandura, 2001), assuming that “people are active agents in shaping their environments” (McClellan, 2017, p. 69), and that individual, behavioral, and environmental characteristics are mutually related to each other (i.e., the triadic reciprocal determinism, Bandura, 2001, 2006), and (2) the personality-psychopathology models discussed by Tackett (2006). Specifically, this study fits the vulnerability/predisposition model and the complication/scar model. The vulnerability/predisposition model posits that an individuals’ premorbid personality may lead to a greater risk to develop psychopathology. For example, in childhood and adolescence, high neuroticism or negative emotionality, as well as low self-efficacy about anger regulation, predict internalizing and externalizing problems (e.g., Gjone and Stevenson, 1997; Di Giunta et al., 2018). The complication/scar model posits that previous psychopathological symptoms may increase some individuals’ predisposition for the risk of psychopathology (Tackett, 2006). For example, former internalizing and externalizing problems were associated with adolescents’ low self-efficacy beliefs about anger regulation and high irritability (Di Giunta et al., 2018, 2020).

Overall, self-efficacy beliefs in dealing with negative emotions could be important determinants of youths’ internalizing and externalizing problems, by acting on many aspects of one’s response to emotional experience, such as their interpretation of situations, strategies to regulate emotions, emotional expressions, and evaluations of consequences (e.g., Bandura and National Institute of Mental Health [NIMH], 1986; Caprara et al., 2013). Low self-efficacy in dealing with negative emotions is associated with anxiety, depression, aggressive, and rule-breaking behaviors (e.g., Bandura et al., 2003; Caprara et al., 2010; Zullig et al., 2014; Valois et al., 2015).

### Self-efficacy in emotion regulation and adjustment in cross-cultural settings

Culture may affect how people experience emotions, assess emotions, and cope with situations that elicit emotion (Markus and Kitayama, 1991; Kitayama et al., 2000; Mesquita, 2001). People from collectivist cultures, compared to people from individualistic cultures, use different strategies to regulate emotions (Matsumoto et al., 2008; Wong, 2009). These cultural differences in emotional experiences and in emotion regulation could also influence the way in which self-efficacy beliefs regarding managing negative emotions develop (Bandura, 2002).

Previous studies examining the cross-sectional and longitudinal associations between self-efficacy regarding anger and sadness regulation, anger and sadness, and (mal)adjustment indicators have found some similarity across cultures (Di Giunta et al., 2017, 2018). Specifically, the direction and the strength of those paths were not significantly different across cultures (including the Colombian and the Italian context). However, we are not aware of previous studies that have examined associations among parenting, negative emotions, and self-efficacy regarding emotion regulation across cross-cultural contexts.

In prior literature relevant to the current study, researchers have found support for the association between positive parenting (and low negative parenting) and youth adjustment (including low emotion regulation difficulties) in a variety of cultural contexts (without testing cross-cultural differences or similarities) such as China (e.g., Bi et al., 2022), Germany (Otterpohl and Wild, 2015), Italy (Di Giunta et al., 2022), Pakistan (Jabeen et al., 2013), and Turkey (Aka and Gencoz, 2014), as well as in different ethnic groups in the U.S. (Valiente and Eisenberg, 2006). Few researchers have examined whether parenting predicts children’s emotional development similarly cross-culturally. For example, in a study of an individualist culture (Australia) and a collectivist culture (Indonesia), greater importance placed on tradition attenuated the positive effect of authoritative parenting on child outcomes (Haslam et al., 2020). In another study (Di Giunta et al., 2018), a cross-culturally invariant mediating role of adolescents’ self-efficacy regarding anger regulation emerged in the association between maternal self-efficacy about anger regulation and adolescents’ internalizing and externalizing problems in Colombia, Italy, and three racial groups in the US. In addition, Di Giunta et al. (2020) found cross-cultural similarities in nine different countries in the mediating role of adolescents’ irritability in the association between harsh parenting and internalizing and externalizing problems. Despite these similarities, other studies suggest cross-cultural differences in the associations between parenting and emotion regulation-related indicators (Trommsdorff and Kornadt, 2003; Neoh et al., 2021).

The aforementioned studies suggest the importance of examining the associations among parenting, emotion regulation-related indicators, and adjustment while also considering the role of the cultural context.

In line with the vast majority of the aforementioned studies, it was hypothesized that parenting would predict adolescents’ self-efficacy about anger and sadness regulation and that the latter would predict late adolescents’ internalizing and externalizing symptoms similarly across countries.

## The present study

The present study had three main aims: (1) examining the growth curves of self-efficacy beliefs regarding anger and sadness regulation from pre- to late-adolescence (from 10 to 18 years old); (2) examining harsh parenting and parental warmth, and children’s internalizing and externalizing problems when children were 10 years old, as predictors of those growth curves; and (3) examining the association of the self-efficacy growth curves with internalizing and externalizing problems at 18 years old. We controlled the growth parameters of self-efficacy beliefs regarding anger and sadness regulation for anger and sadness when children were 12 years old. We then examined whether those paths were similar or different in Colombia and Italy.

In line with the previous study showing a growth curve of self-efficacy in regulating overall negative emotions that did not change from 15 to 21 years old (Caprara et al., 2013), we expected a similar trend for self-efficacy in regard to both anger and sadness regulation. In line with previous studies (Niditch and Varela, 2012; Bi et al., 2022; Di Giunta et al., 2022; Liu et al., 2022), high parental warmth and low harsh parenting were expected to be associated with high initial levels of the growth curves for both self-efficacy regarding anger and sadness regulation, as well as with an increase in those self-efficacy beliefs over the adolescent transition. In addition, consistent with prior related findings (e.g., Bandura et al., 2003; Caprara et al., 2010; Di Giunta et al., 2018, 2022), we predicted a negative association between high initial levels of the growth curves of self-efficacy regarding anger and sadness regulation and low internalizing and externalizing problems (when controlling for initial levels of internalizing and externalizing). We also predicted that increasing slopes of both forms of self-efficacy would predict lower levels of symptoms of the two forms of psychopathology.

These predictions were examined using data from two countries, and controlling for child gender and parental education, although the analyses regarding culture were exploratory.

## Materials and methods

### Participants

Participants were part of the larger study entitled Parenting Across Cultures (PAC; e.g., Lansford et al., 2018). A normative sample of adolescents (*N* = 285; T1: *M*_*age*_ = 10.57, *SD* = 0.68; 53.3% female; T2: *M*_*age*_ = 12.10, *SD* = 1.09; T3: *M*_*age*_ = 13.33, *SD* = 1.25; T4: *M*_*age*_ = 14.06, *SD* = 1.11; T5: *M*_*age*_ = 15.65, *SD* = 0.70; T6: *M*_*age*_ = 18.45, *SD* = 0.71) and their parents (mothers *n* = 286; fathers *n* = 276) participated to the study. Families were recruited from Medellín, Colombia, and Naples and Rome, Italy. Supplementary Table 1 reports sample sizes for both countries, separately for parents and adolescents, at each time-point. Adolescent participation rates were high across times (i.e., 89–98%). Supplementary Table 2 summarizes marital status and parents’ years of education for both countries.

### Procedure

Following Institutional Review Board protocol in each country, once informed consent was obtained, participants were enrolled in each country until target sample sizes were reached. Participants were recruited from diverse schools with high-, middle-, and low-income families, approximately matching the socio-economic stratification of the population of each site. Measures were administered in the predominant language of the family (Spanish in Colombia; Italian in Italy). We used translation and back-translation to guarantee the conceptual and linguistic equivalence of instruments across languages (Maxwell, 1996). Interviews were conducted in participants’ homes or other preferred location. Each interview lasted approximately 1 h. Participants were given modest financial compensation.

### Measures

All measures were youth-reported except for parental years of education (T1) and youths’ anger and sadness (T2). All measures have been validated in previous studies including Colombian and Italian samples (e.g., Di Giunta et al., 2018, 2020, 2022; Lansford et al., 2018; Rothenberg et al., 2020, 2022; Lunetti et al., 2022).

#### Demographic variables

Child gender (0 = boys, 1 = girls) and parent-reported years of parental education at T1 were included as covariates.

#### Parental warmth (T1)

Youths completed 8 items derived from the Parental Acceptance-Rejection/Control Questionnaire (Short Form) (Rohner, 2005) for the assessment of maternal warmth (1 = *almost never*, 4 = *every day*; e.g., “My mother let me know she loves me”; α = 0.72 and 0.74, respectively for Colombia and Italy) and 8 items for the assessment of paternal warmth (α = 0.83 and 0.85, respectively for Colombia and Italy). Youth-reported maternal and paternal warmth were strongly correlated with each other (*r* = 0.60; *p* < 0.001), therefore, those scores were averaged into a composite score of parental warmth.

#### Harsh parenting (T1)

Harsh parenting was measured with 7 items from the Discipline Interview, which has demonstrated excellent reliability and validity in numerous cultures worldwide, including the countries included in the current study (Huang et al., 2011). Youths were asked the frequency (1 = *never;* 5 = *almost every day*) with which their mothers and fathers used 7 different harsh discipline behaviors (e.g., spanking, shaming, yelling at). Item scores were averaged to create the score for harsh parenting. Higher score indicated harsher parenting. In the current study, the scale demonstrated good internal consistency across countries for youth-reported maternal (α = 0.70 and 0.76, respectively for Colombia and Italy) and paternal (α = 0.70 and 0.77, respectively for Colombia and Italy) harsh parenting. Youth-reported maternal and paternal harsh parenting were strongly correlated to one other (*r* = 0.64; *p* < 0.001); therefore, those scores were averaged into a composite score of harsh parenting.

#### Youths’ externalizing and internalizing problems (T1; T6)

At T1, youths completed Achenbach’s (1991) Youth Self-Report. At T6 youths completed the Adult Self-Report (ASR; Rescorla and Achenbach, 2004). At both T1 and T6, participants were asked to rate how true each item was during the last 6 months (0 = *not true*, 1 = *somewhat or sometimes true*, 2 = *very or often true*). The *Externalizing Behavior* scale was the average across 30 items (YSR; α = 0.74 and 0.78, respectively for Colombia and Italy) and 26 items (ASR; α = 0.90 and 0.83, respectively for Colombia and Italy) and captured behaviors such as lying, truancy, vandalism, bullying, drug and alcohol use, disobedience, tantrums, sudden mood change, and physical violence. The *Internalizing Behavior* scale was the average across 29 items (YSR; α = 0.81 and 0.84, respectively for Colombia and Italy) and 25 items (ASR; α = 0.90 and 0.91, respectively for Colombia and Italy) and measured behaviors and emotions such as loneliness, self-consciousness, nervousness, sadness, and anxiety. The Achenbach measures are among the most widely used instruments in international research, with translations in over 100 languages and strong, well-documented psychometric properties (e.g., Achenbach and Rescorla, 2006).

#### Youths’ anger and sadness (T2)

Mothers completed 17 negative emotionality items (9 items for anger and 8 items for sadness) on the Early Adolescent Temperament Questionnaire-Revised (EATQ-R; Capaldi and Rothbart, 1992), indicating how well statements described their child (1 = *almost always untrue*; 5 *almost always true*). Anger (e.g., “Gets very irritated when someone criticizes him/her”) and sadness (e.g., “More sad than others realize”) items were averaged to create a composite score for anger and sadness (α anger = 0.88 and 0.85, respectively for Colombia and Italy and α sadness = 0.79 and 0.73, respectively for Colombia and Italy). Previous studies have supported the psychometric properties of this instrument in a variety of cultural groups (e.g., Capaldi and Rothbart, 1992; Viñas Poch et al., 2015; Esposito et al., 2017).

#### Youths’ self-efficacy beliefs in regard to anger and sadness regulation (from T2 to T6)

Youths rated (1 = *not well at all*; 5 = *very well*) how well they believed they were able to manage anger and sadness with 3 items, respectively (e.g., self-efficacy about anger regulation: “How well can you avoid flying off the handle when you get angry?”; mean α across countries and time-points = 0.81; self-efficacy about sadness regulation: “How well can you keep from getting discouraged in the face of difficulties?”; mean α across countries and time-points = 0.78) from the Regulative Emotional Self-Efficacy Scale (Caprara et al., 2008). Higher scores indicate greater self-efficacy in regard to anger and sadness regulation.

### Data analytic approach

Latent Growth Curve Modeling (LGCM) adjusted for unequal time points with maximum-likelihood estimation was implemented in M*Plus* 8 (Muthén and Muthén, 2017) to assess first, the development of youths’ self-efficacy beliefs regarding anger regulation (Model 1) and then, youths’ self-efficacy beliefs regarding sadness regulation (Model 2) in the full sample. In both models two latent factors were estimated: (1) the intercept, representing initial levels of youths’ self-efficacy beliefs at T2 and (2) the slope, representing the rate of change in self-efficacy beliefs over time (from T2 to T6). To identify the best fitting trajectory, we tested three unconditional models: a random-intercept only no growth model (i.e., strict stability or no growth model, including only the intercept; mean and variance of the slope are supposed to be zero), a linear growth model (i.e., representing a constant change over time; the mean and the variance of the slope are freely estimated), and a quadratic growth model with two latent factors of change estimates, namely, the linear and quadratic trends. A model was considered to have good fit if the χ^2^ test was non-significant (*p* ≥ 0.05), the CFI ≥ 0.95, the RMSEA ≤ 0.06, and the SRMR ≤ 0.08 (Hu and Bentler, 1999). Because models were nested, we performed a chi-square difference test (Δχ^2^) to identify the best fitting model (Kline, 2010).

We then assessed possible cultural differences in the development of youths’ self-efficacy beliefs regarding anger and sadness regulation using multi-group analyses (e.g., Rothenberg et al., 2020). We estimated an unconstrained model where *no* parameters were constrained to be equal across groups and compared this model to a model where *all* structural paths were constrained to be equal across groups. If the Δχ^2^ between the constrained and unconstrained multi-group models was significant (*p* < 0.05), we examined modification indices to release paths that differed across groups (Cheung and Rensvold, 2002). The final model from these analyses was used as our baseline model when examining predictors and the outcomes of self-efficacy growth.

To examine how well parenting, anger, and their interactions predicted initial levels and growth in youths’ self-efficacy beliefs regarding anger regulation, and how much self-efficacy regarding anger regulation predicted youths’ internalizing and externalizing problems cross-culturally (Model 1), we ran a conditional multi-group model in which we added: (a) as predictors: T1 parental warmth and harsh parenting, T2 youths’ anger and the interactions between parental warmth and youths’ anger and between harsh parenting and youths’ anger; (b) as outcomes: T6 youths’ internalizing problems and T6 youths’ externalizing problems. We also controlled for the stability of the considered outcomes; thus, we added as predictors T1 youths’ internalizing and externalizing problems. Moreover, youths’ gender and parental educational level were treated as covariates and their impact on all the study variables was examined.

Similarly, in Model 2, we examined the effects of parenting, youths’ sadness and their interactions on the initial levels and growth in youths’ self-efficacy beliefs regarding sadness regulation, as well as how much self-efficacy beliefs regarding sadness regulation predicted youths’ internalizing and externalizing problems cross-culturally. Specifically, we ran our conditional multi-group model in which we added: (a) as predictors: T1 parental warmth and harsh parenting, T2 youths’ sadness and the interactions between parental warmth and youths’ sadness and between harsh parenting and youths’ sadness; (b) as outcomes: T6 youths’ internalizing problems and T6 youths’ externalizing problems; and (c) as covariates: youths’ gender and parents’ educational level. Moreover, we controlled for the stability of the outcomes by adding T1 youths’ internalizing and externalizing problems as predictors.

Last, we ran a multi-group conditional LGCM to examine potential differences between Colombia and Italy in how predictors and interaction terms were associated with changes in self-efficacy beliefs, and how these changes were associated with youths’ internalizing and externalizing problems.

## Results

### Descriptive statistics and correlations

Supplementary Table 3 reports means, standard deviations, skewness, and kurtosis for variables from T1 to T6 within the overall sample. Correlations among variables within the overall sample are in Supplementary Tables 4, 5. Descriptive statistics and correlations separately by country are in Supplementary Tables 6–10.

### Conditional LGCMs–Model 1: anger-related variables

**FIGURE 1:**
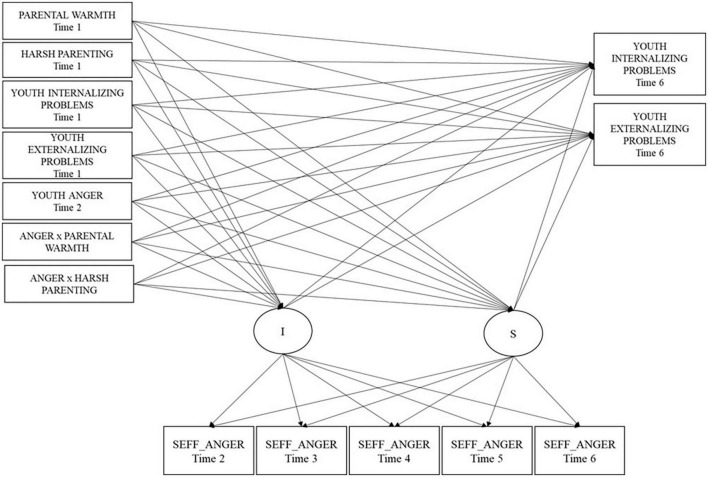
Conditional multi-group latent growth curve model 1 for self-efficacy about anger regulation for the two cultural groups. We also estimated the correlations within the time among predictors and outcomes and the effect of covariates (youths’ gender and parents’ educational level. SEFF_Anger refers to self-efficacy in anger regulation.

**FIGURE 2:**
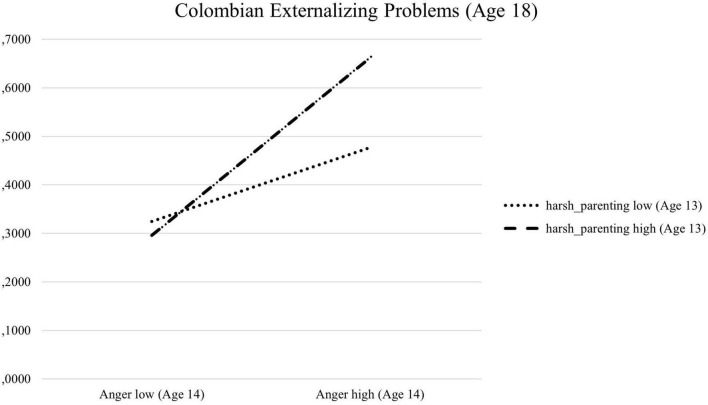
Interaction effect of T1 harsh parenting and T2 anger in predicting T6 externalizing problems on the Colombian sample.

Table 1 reports the results of unconditional and conditional multi-group LGCMs across Colombia and Italy for Model 1. Unconditional LGCMs indicated the linear model as the best- fitting model, with only one parameter different across cultures, which was the mean of the initial levels of self-efficacy beliefs regarding anger regulation (Italy = 3.03; Colombia = 3.30).^1^ Therefore, we ran our conditional multi-group model as described in the analytical approach section. To guarantee model parsimony (Rothenberg et al., 2020), we excluded the non-significant within T1 correlations among predictors (9 correlations), and the non-significant effects of covariates (11 effects). This final conditional, multi-group, partially constrained model (Figure 1) fit the data moderately well [χ^2^(181) = 248.48, *p* < 0.001, RMSEA = 0.05 (90% CI 0.03, 0.06), CFI = 0.92, TLI = 0.90, SRMR = 0.08] and was not statistically different from the correspondent model in which the effects of covariates and the within-time correlations were fully unconstrained across Colombia and Italy [Δχ^2^(54) = 26.27, *p* = 0.99]. In this final model, we incrementally released 5 within-time correlations among predictors; 2 effects of covariates on some predictors; and 1 effect of T1 youths’ internalizing problems on T6 youths’ internalizing problems (−0.02; *p* = 0.89 in Colombia; 0.38; *p* < 0.01 in Italy). The parameters to be different in the unconditional model were the mean of the intercept, and the interaction between harsh parenting and youths’ anger in predicting T6 youths’ externalizing problems, which was significant only in the Colombian group (0.23; *p* < 0.001 in Colombia; 0.05; *p* = 0.17 in Italy). This statistically significant, culturally variant interaction effect was explored *post hoc* by plotting values of the outcome’s levels at high and low (under and above the median) anger and harsh parenting (Figure 2). Youths who were high in T2 anger and who also experienced high T1 harsh parenting were higher in T6 externalizing problems than those youths who were low in T2 anger and low in T1 harsh parenting.

**TABLE 1:** Unconditional and conditional multi-group latent growth curve models 1 for self-efficacy about anger regulation.

	Estimate^a^	S.E.	*P*-value
**Conditional model**			
Intercept with slope^b^	−0.01	0.01	0.179
Intercept variance	0.19	0.04	<0.001
Intercept mean	2.91^c^; 3.07^d^	0.05^c^;0.50^d^	<0.001^c^; <0.001^d^
Slope variance	0.01	0.003	0.001
Slope mean	2.81	0.13	0.027
**Predictors of intercept**			
T1 Parental warmth	0.21	0.11	0.064
T1 Harsh parenting	−0.17	0.08	0.045
T1 Youth internalizing problems	0.14	0.21	0.495
T1 Youth externalizing problems	−0.77	0.28	0.006
T2 Youth anger	−0.07	0.05	0.175
Parental warmth*anger	0.09	0.14	0.496
Harsh parenting*anger	−0.14	0.09	0.123
**Predictors of linear slope**			
T1 Parental warmth	−0.05	0.02	0.115
T1 Harsh parenting	0.03	0.02	0.225
T1 Youth internalizing problems	−0.06	0.06	0.300
T1 Youth externalizing problems	−0.02	0.07	0.765
T2 Youth anger	−0.05	0.01	<0.001
Parental warmth*anger	−0.04	0.04	0.289
Harsh parenting*anger	0.04	0.02	0.057
**Effects on youth internalizing problems**			
Intercept	−0.21	0.06	0.001
Slope	−0.70	0.28	0.016
T1 Parental warmth	−0.03	0.06	0.616
T1 Harsh parenting	−0.001	0.05	0.997
T1 Youth internalizing problems	−0.02^c^;0.38^d^	0.17^c^;0.13^d^	0.896^c^;0.002^d^
T1 Youth externalizing problems	−0.15	0.16	0.352
T2 youth anger	−0.003	0.03	0.918
Parental warmth*anger	0.05	0.08	0.550
Harsh parenting*anger	0.07	0.05	0.156
**Effects on youth externalizing problems**			
Intercept	−0.15	0.04	0.001
Slope	−0.17	0.08	<0.001
T1 Parental warmth	0.04	0.04	0.406
T1 Harsh parenting	0.05	0.03	140
T1 Youth internalizing problems	−0.12	0.08	0.126
T1 Youth externalizing problems	0.39	0.11	<0.001
T2 Youth anger	−0.01	0.02	0.731
Parental warmth*anger	0.08	0.05	0.123
Harsh parenting*anger	0.23^c^;0.05^d^	0.06^c^;0.04^d^	<0.001^c^;0.174^d^

^a^Estimates are unstandardized betas unless otherwise indicated.

^b^Estimate is a correlation coefficient.

^c^Colombia.

^d^Italy.

*Interaction term.

In the final conditional, multi-group, partially constrained model, the mean intercept which was different across groups, was slightly higher in Italy (3.07) than in Colombia (2.91); the mean slope was significant, positive, and similar across groups (2.81; *p* < 0.01), suggesting an overall linear increasing trajectory of self-efficacy beliefs in regard to managing anger for both groups. Also, the variances of the intercept (0.19; *p* < 0.001) and slope (0.01; *p* < 0.001) were similar across cultures and significant (i.e., there was significant difference across individuals in the initial level of the growth curve of self-efficacy in anger regulation and in its trend over time). The unstandardized estimates of within-time correlations among predictors and covariate effects in the final conditional, multi-group, partially constrained model are presented separately by cultural group in Supplementary Tables 11, 12. The details of the unstandardized estimates of growth parameters and the relation of predictors and outcomes to growth parameters are reported in Table 1.

T1 harsh parenting and T1 youths’ externalizing problems negatively predicted initial levels of self-efficacy beliefs regarding anger regulation similarly across countries, suggesting that previous high harsh parenting and high externalizing problems were related to low initial levels of self-efficacy beliefs. In terms of the relation of the predictors to the slope of the growth curve of self-efficacy beliefs regarding anger regulation, the negative relation of youths’ anger to the slope was similar across groups, suggesting that, both in Colombia and Italy, high previous levels of youths’ anger were associated with less increase in self-efficacy beliefs regarding anger regulation over time. Regarding prediction of growth factors on youths’ later internalizing and externalizing outcomes, high initial levels of self-efficacy beliefs regarding anger regulation were associated with low subsequent internalizing and externalizing problems. Similarly, a greater increase of self-efficacy beliefs in anger regulation was associated with youths’ low later internalizing and externalizing problems.

### Conditional LGCMs–Model 2: sadness-related variables

**FIGURE 3:**
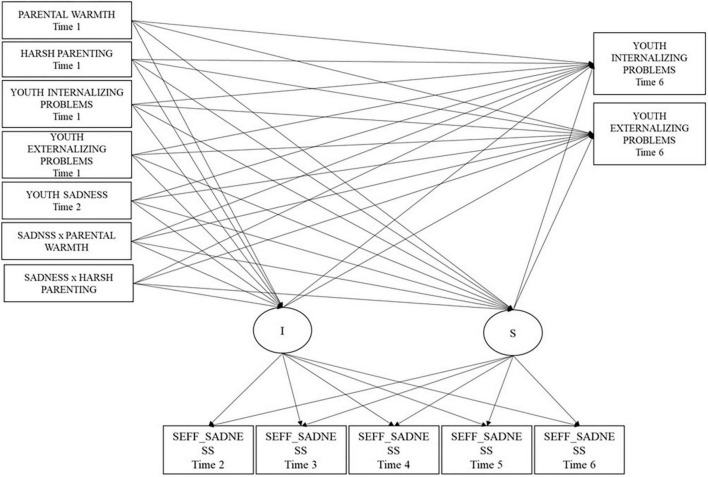
Conditional multi-group latent growth curve model 2 for self-efficacy about sadness regulation for the two cultural groups. We also estimated the correlations within the time among predictors and outcomes and the effect of covariates (youths’ gender and parents’ educational level. SEFF_Sadness refers to self-efficacy in sadness regulation.

Table 2 reports the results of unconditional and conditional multi-group LGCMs across Colombia and Italy. Furthermore, to guarantee model parsimony (Rothenberg et al., 2020), we excluded the non-significant within T1 correlations among predictors (6 correlations), and the non-significant effects of covariates (11 effects). This final conditional, multi-group, partially constrained model (Figure 3) fit the data well [χ^2^(180) = 224.57, *p* < 0.001, RMSEA = 0.04 (90% CI 0.02, 0.05), CFI = 0.94, TLI = 0.92, SRMR = 0.09] and was not statistically different from the correspondent model in which the effects of covariates and the within-time correlations were fully unconstrained across Colombia and Italy [Δχ^2^(47) = 61.27, *p* = 0.78].

**TABLE 2:** Unconditional and conditional multi-group latent growth curve models 2 for self-efficacy about sadness regulation.

	Estimate^a^	S.E.	*P*-value
**Conditional model**			
Intercept with slope^b^	−0.01	0.01	0.485
Intercept variance	0.15	0.03	<0.001
Intercept mean	3.15	0.46	<0.001
Slope variance	0.01	0.003	0.101
Slope mean	0.06	0.12	0.599
**Predictors of intercept**			
T1 Parental warmth	0.17	0.11	0.109
T1 Harsh parenting	−0.04	0.08	0.603
T1 Youth internalizing problems	0.05^c^;−0.83^d^	0.26^c^;0.21^d^	0.846^c^;<0.001^d^
T1 Youth externalizing problems	0.24	0.26	0.368
T2 Youth sadness	−0.23^c^;−0.09^d^	0.07^c^;0.06^d^	<0.001^c^;0.141^d^
Parental warmth* sadness	0.07	0.17	0.665
Harsh parenting* sadness	−0.04	0.13	0.776
**Predictors of linear slope**			
T1 Parental warmth	0.01	0.02	0.627
T1 Harsh parenting	0.02	0.03	0.453
T1 Youth Internalizing Problems	0.06	0.05	0.251
T1 Youth externalizing problems	−0.08	0.07	0.259
T2 Youth sadness	0.000	0.02	0.984
Parental warmth* sadness	−0.03	0.05	0.493
Harsh parenting* sadness	−0.001	0.04	0.985
**Effects on youth internalizing problems**			
Intercept	−0.19	0.12	0.021
Slope	−0.09	0.03	0.055
T1 Parental warmth	0.01	0.08	0.861
T1 Harsh parenting	0.07	0.07	0.324
T1 Youth internalizing problems	0.35	0.18	0.046
T1 Youth externalizing problems	−0.14	0.22	0.543
T2 Youth sadness	0.04	0.04	0.343
Parental warmth* sadness	−0.01	0.14	0.926
Harsh parenting* sadness	−0.03	0.10	0.784
**Effects on youth externalizing problems**			
Intercept	0.07	0.07	0.314
Slope	−0.68	0.80	0.293
T1 Parental warmth	0.05	0.05	0.199
T1 Harsh parenting	0.08^c^;0.05^d^	0.04^c^;0.04^d^	0.061^c^;0.228^d^
T1 Youth internalizing problems	0.07	0.10	0.518
T1 Youth externalizing problems	0.48	0.13	<0.001
T2Youth sadness	0.05	0.03	0.061
Parental warmth* sadness	0.05	0.08	0.566
Harsh parenting* sadness	−0.01	0.06	0.925

^a^Estimates are unstandardized betas unless otherwise indicated.

^b^Estimate is a correlation coefficient.

^c^Colombia.

^d^Italy.

*Interaction term.

Overall, in this final model, we incrementally released 7 within-time correlations among predictors, 2 effects of covariates on some predictors, 1 effect of T1 harsh parenting on T6 youths’ externalizing problems (−0.08; *p* = 0.06 in Colombia; 0.05; *p* = 0.23 in Italy), the effects of T1 youths’ internalizing problems on the intercept of self-efficacy beliefs about regulating sadness (which was significant only in Italy; 0.05; *p* = 0.85 in Colombia; −0.83; *p* < 0.001 in Italy), and the effect of T2 youths’ sadness on the intercept of self-efficacy beliefs in regulating sadness (which was significant only in Colombia; −0.23; *p* < 0.001 in Colombia; −0.09; *p* = 0.14 in Italy).

In the final conditional, multi-group, partially constrained model, the mean intercept was similar across groups and was positive (3.15); the mean slope was not significant (0.06; *p* = 0.59) and this result was similar across cultures, suggesting a trajectory of self-efficacy beliefs in sadness regulation that did not change over time for both groups. Also, the variance of the intercept was similar across cultures and significant (0.15; *p* < 0.001; i.e., there was significant difference across individuals in the initial level of the growth curve of self-efficacy in sadness regulation), whereas the slope’s variance was similar across cultures and not significant (0.01; *p* = 0.10; i.e., there was not significant difference across individuals in the trend of self-efficacy in sadness regulation over time). The unstandardized estimates of within-time correlations among predictors and covariate effects from the final conditional, multi-group, partially constrained model are in Supplementary Tables 13, 14, separately by country. The unstandardized estimates of the growth parameters and the relation of predictors and outcomes to growth parameters are reported in Table 2. In terms of the relation of the predictors to the intercept of the growth curve of self-efficacy beliefs, T1 youths’ internalizing problems negatively predicted initial levels of self-efficacy beliefs regarding sadness regulation only in Italy, suggesting that in this cultural context, higher previous levels of internalizing problems were associated with lower self-efficacy beliefs regarding sadness regulation, and T2 youths’ sadness negatively predicted the intercept of self-efficacy beliefs regarding sadness regulation only in Colombia, suggesting that in this cultural context, previous higher levels of youths’ sadness were associated with lower initial levels of self-efficacy beliefs regarding sadness regulation. No predictors significantly predicted the slope. Lastly, regarding the effects of intercept and slope on the outcomes, only the intercept negatively predicted youths’ T6 internalizing problems in both countries.

## Discussion

The present longitudinal, cross-cultural study contributes to knowledge about the development of self-efficacy beliefs in the domain of emotion regulation, and in particular, self-efficacy beliefs about anger and sadness regulation across adolescence. This study’s goals were four-fold: (1) to examine the growth curves of self-efficacy regarding anger and sadness regulation, respectively, from 12 to 18 years old: (2) to examine prediction of growth parameters of the aforementioned curves (i.e., initial level and slope by antecedent family predictors, namely, harsh parenting and parental warmth when children were 10 years old) and individual predictors (i.e., internalizing and externalizing problems when children were 10 years old; anger and sadness when they were 12 years old); (3) to examine the predictive role of the growth parameters on maladaptive outcomes at 18 years old (i.e., internalizing and externalizing problems); and (4) to examine all the aforementioned aims in a cross-cultural perspective by comparing results in Colombia and Italy. All aims were examined using child gender and parental education as covariates.

Unconditional (without any predictors, outcomes, and covariates) and conditional (including predictors, outcomes, and covariates) multi-group, latent growth curve modeling across Colombia and Italy was used to examine growth curves. The final results of the conditional models are summarized in order to highlight the most comprehensive, fine-grained picture of how self-efficacy beliefs regarding anger and sadness regulation develop, accounting for predictors that contribute to that developmental trend, and discussing outcomes associated with that developmental trajectory. For sake of clarity, a summary of the results and a discussion of results from the *all-in-one* conditional models are divided in sub-sections related to (a) the developmental trends of self-efficacy regarding anger and sadness regulation; (b) the association of those curves with predictors and outcomes; and (c) the cross-cultural similarities and differences that emerged in those results.

### Developmental growth curve for self-efficacy regarding anger and sadness regulation

In the final conditional, multi-group, partially constrained model, similar across the two groups, over time youths believed themselves to be increasingly more competent in managing their anger in the face of difficulties and challenging situations. Moreover, Italians reported being more capable than Colombians at dealing with anger at 12 years old. In regard to growth in self-efficacy regarding the regulation of sadness, the trajectory did not change over time, and the initial level and slope did not differ significantly across cultures.

The variances of the intercept and slope for self-efficacy regarding anger regulation, as well as the intercept for self-efficacy regarding sadness regulation, were significant in both cultural contexts. In contrast, overall, both for Colombia and Italy, the variance of the slope for self-efficacy regarding sadness regulation was not statistically significant (i.e., it was similar between the children in the present study). Future studies might examine the trajectories or growth mixture models of self-efficacy regarding emotion regulation to further explore the existence of sub-groups of youths with different initial levels of self-efficacy regarding anger and sadness regulation and/or different developmental trends over time.

The developmental patterns that emerged cross-culturally for both self-efficacy regarding anger and sadness regulation across adolescence are partially in line with a previous study that examined the growth curve of self-efficacy in regulating overall negative emotions (Caprara et al., 2013). Thenon-changing growth curve for self-efficacy regarding sadness regulation is consistent with the findings of Caprara et al. (2013), whereas the increasing trajectory for self-efficacy regarding anger regulation is not. It might be that the trajectory that does not change over time identified in Caprara et al. (2013) hides different trajectories associated with self-efficacy in regulating different negative emotions. In addition, Caprara et al. (2013) targeted a phase of life that is later than the one targeted in the present study, namely, the transition from adolescence to young adulthood in the former, and the transition from pre- to late-adolescence in the present study. Perhaps the increasing trajectory for self-efficacy regarding anger regulation identified in the present study predates developmentally the trajectory found in Caprara et al. (2013). However, this is speculation that merits examination in future studies capitalizing on a longer longitudinal study extending from childhood to young adulthood.

Given the associations in other studies of high self-efficacy regarding anger regulation with low anger and low anger dysregulation, as well as associations of high self-efficacy regarding sadness regulation with low sadness and low sadness dysregulation (e.g., Di Giunta et al., 2017), it might be worth examining the correspondence between developmental trends of emotion-related self-efficacy and of emotional experience. In particular, the decreasing trajectory of anger in the adolescent transition reported in Maciejewski et al. (2017) might be considered consistent with the increasing trend that emerged for self-efficacy regarding anger regulation in the present study. However, this same reasoning is not applicable in the case of sadness; indeed, the increasing sadness growth curve in the adolescent transition reported in Maciejewski et al. (2017) is not consistent with the non-changing curve over time of self-efficacy regarding sadness regulation that emerged in the present study. It might be speculated that, despite a normative period characterized by increasing trend of sadness over time, whether children believe themselves to be capable of handling sadness develops relatively early and does not change over time. In addition, considering that the most powerful source of self-efficacy development is modeling (Bandura, 1997), the stability of self-efficacy regarding sadness regulation may show that the source might not be so powerful in this specific domain of self-efficacy. Indeed, this result shows a higher impact that personal/temperamental factors (e.g., sadness), rather than environmental factors (e.g., parenting and opportunities of being exposed to modeling of self-efficacy), have on its development (see results reported below about the impact of predictors on the growth curve of self-efficacy regarding sadness regulation). Certainly, those speculations deserve further exploration. Future studies could examine the joint curves of both negative emotionality and self-efficacy in emotion regulation over time.

### The association of predictors and outcomes with the growth curves of self-efficacy regarding anger and sadness regulation

Regarding the association between parenting-related predictors and the growth parameters in self-efficacy beliefs about both anger and sadness regulation, when pre-adolescents reported that their parents tended to yell, scold, and use threats as discipline and when they reported behaving aggressively in late childhood, pre-adolescents tended to report they were less capable of handling anger in frustrating, difficult, challenging situations. There was not a significant association between parenting and the increasing trend of self-efficacy growth curves. The lack of association between the family predictors examined in this study and both the initial level and the slope of the non-changing curve of self-efficacy about sadness regulation might indicate that when youths think whether and how they could be capable of dealing with their sadness, they are rather uninfluenced by family factors that could potentially impact those beliefs. Maybe the beliefs associated with the specific emotion of sadness are more affected by individual factors than contextual ones. Conversely, in the event youths think whether and how they could be capable of dealing with their anger, there are family factors that can improve or buffer the initial levels of those beliefs, but they are not associated with their development over time. It may also be that there are other family factors that were not examined in the present study, but that may potentially be associated with those youths’ beliefs (e.g., correspondent beliefs in parents; Di Giunta et al., 2018). It might also be that the initial level of self-efficacy about anger and sadness regulation could mediate the association between parenting and their growth over time. Those are all hypotheses that go beyond the goals of this study, and future studies should focus on exploring them.

Moreover, children who were relatively high in dispositional anger tended to increase less over time in their beliefs they were capable of handling anger across adolescence. These findings did not differ across cultural groups. No interactions between parenting and anger predicted growth parameters of self-efficacy about anger regulation. Moreover, similarly across cultures, a higher level of self-efficacy regarding anger regulation at 12 years old, as well as an increase in self-efficacy regarding anger regulation across adolescence, was associated with fewer internalizing and externalizing problems in late adolescence (and these findings did not differ across cultures). Thus, both parenting and youths’ emotionality predicted their self-efficacy in predictable ways and similarly across youths on two different continents.

Unexpectedly, a relatively high level of internalizing problems at 10 years old was significantly associated with lower self-efficacy beliefs regarding sadness regulation at 12 years old only in the Italian context. In the Colombian context, higher sadness at age 12 old was significantly associated with lower self-efficacy beliefs regarding sadness regulation at 12 years old. An increasing slope of self-efficacy about sadness regulation did not predict lower levels of problems in late adolescence, likely because the beliefs youths held about their capacity to handle sadness in sad and challenging situations tend not to change over the adolescent transition. It is not whether youths increase those specific beliefs, but whether they believed themselves to be capable of dealing with sadness in discouraging situations at 12 years old that was associated with low anxiety-depressive and somatic symptoms, and social withdrawal at 18 years old, and this finding did not vary across the two cultures.

The associations that emerged between predictors and outcomes and the developmental growth curves of self-efficacy in anger and sadness regulation are overall in agreement with previous studies. Those associations also supported the protective role of low negative parenting and low negative emotionality in promoting adaptive personality/emotional development in adolescence (e.g., Steinberg et al., 1994; Niditch and Varela, 2012; Bi et al., 2022). In addition, the predictive role of the development of self-efficacy about anger and sadness regulation on later internalizing and externalizing problems is in line with those studies supporting the association between higher self-efficacy in emotion regulation and fewer socio-emotional difficulties (Bandura et al., 2003; Caprara et al., 2010; Di Giunta et al., 2017, 2020, 2022; Liu et al., 2022; Lunetti et al., 2022). The reciprocal relation between self-efficacy in anger and sadness regulation and fewer problematic behaviors that emerged in the present study is in agreement with the tenets of triadic reciprocal determinism, regarding reciprocal influences between individual, behavioral, and contextual characteristics (Bandura, 2001, 2006). Moreover, the association between self-efficacy and later low problematic behaviors is also in line with the vulnerability model of personality-psychopathology framework developed by Tackett (2006), in which specific characteristics of personality can predispose the person to develop psychopathology. The association between low problematic behaviors and later improvement in self-efficacy is in line with the scar model (Tackett, 2006), which posits that early problematic behaviors may undermine healthy personality development over time which, in turn, might contribute to the risk of subsequent psychopathology. That said, it is important to keep in mind that participants from both countries in this study were normative samples and not clinical ones. Thus, future studies could verify whether the results of the present study are corroborated with longitudinal study targeting clinical samples of adolescents.

Family factors significantly predicted the development of self-efficacy regarding anger regulation, but not the development of self-efficacy regarding sadness regulation, whereas individual factors were similarly predictive of the development of self-efficacy regarding anger and sadness regulation. These results support the view that the development of self-efficacy regarding anger regulation is influenced by both individual and environmental factors, whereas the development of self-efficacy beliefs regarding sadness regulation seems to be either mainly constitutionally based or might be influenced by other parenting (or contextual) factors that were not considered in the present study. Perhaps the management of anger is more salient to adults than the modulation of sadness and, consequently, is more often a target of socialization. Moreover, because sadness may be less observable than anger, it might be less associated with parenting quality. In contrast, adults may attend to children’s anger and the quality of their parenting behavior is likely affected to some degree by children’s display of anger and their ability to manage the manifestations of anger. Such associations could translate into children’s beliefs about their abilities to manage anger and sadness. Overall, the findings suggest there is a need to learn a lot more about factors that account for individual differences in children’s emotionality and their beliefs regarding their abilities to manage these negative emotions.

### Cross-cultural similarities and differences

Our findings regarding the correlates and developmental trajectories of self-efficacy about the regulation of negative emotions, as well as prediction by parenting of self-efficacy, did not differ across the two cultural groups (e.g., Di Giunta et al., 2017, 2022). However, some cultural differences between the Colombian and the Italian samples emerged when considering the role of individual and behavioral factors on the development of self-efficacy. Specifically, in the model that examined the developmental growth of self-efficacy in anger regulation, predictors, outcomes, and covariates, 12-year-old Italians believed themselves to be more capable at anger regulation than did their Colombian peers. Moreover, in Colombia but not Italy more externalizing problems at 18 years old were predicted by higher levels of both harsh discipline and anger at a younger age. Furthermore, in the model that examined the growth curves of self-efficacy in sadness regulation, more internalizing problems at 10 years old predicted lower self-efficacy regarding sadness regulation in Italy, but not in Colombia, and high sadness at 10 years old predicted lower self-efficacy in sadness regulation in Colombia, but not in Italy. Rather than speculating on why those cultural differences could have occurred, we would like to reiterate that the goal of examining cross-cultural similarities or differences in the present study’s paths was exploratory.

Overall, a general pattern of consistency in the findings across the two cultural groups in this study emerged, which can be interpreted as support for the view that Colombian and Italian cultures share, for example, some collectivistic values, such as the importance given to family, in terms of attachment, loyalty, and reciprocity among family members (e.g., Manzi et al., 2006; Velásquez et al., 2006). However, the few cultural differences that emerged in the present study could reflect the fact that Colombia and Italy also differ with regard to a number of factors, such as (a) geography and economics (Organisation for Economic Co-operation and Development [OECD], 2023a,b), (b) family income and number of children in the household that may impact the level of individualism and collectivism (Oyserman et al., 2002) within each country (Lansford et al., 2021), as well as (c) in how adolescents’ self-regulation is linked to risk-taking (Duell et al., 2016). Thus, it is very hard to speculate about the factor(s) that might account for the few cultural differences that emerged in the present study. The development of self-efficacy in the domain of emotion regulation in Colombia might be more susceptible to environmental influences (e.g., parenting), whereas in Italy it might be more susceptible to individual influences (e.g., internalizing problems). However, this is mere speculation that deserves further examination. Future studies could include cultural norms and values that could guide the interpretation of potential cultural differences.

Future studies have to consider additional culturally sensitive variables beyond the examined ones, to clarify, for example, why Italian teenagers might perceive themselves more capable in dealing with anger than Colombians, or why in Colombia individual factors were more important in personality development than in Italy, or why in Italy family factors are more important in personality development than in Colombia.

Overall, the cultural differences that emerged in the present study support the view that multiple mechanisms (cultural, familial, individual) work together in supporting or obstructing healthy personality and socio-emotional development during adolescence. This perspective is in line with cross-cultural psychologists who have argued that culture affects the way people experience emotions, assess emotions, and cope with situations that elicit emotion (Markus and Kitayama, 1991; Kitayama et al., 2000; Mesquita, 2001), which in turn might affect their engagement in specific forms of discipline (Eisenberg et al., 1998), their children’s emotion regulation development (Trommsdorff and Kornadt, 2003; Louie et al., 2013; Haslam et al., 2020; Neoh et al., 2021), and youths’ self-efficacy beliefs regarding emotion regulation.

### Strengths, weaknesses, and implications

There are several clear strengths of this study. First, this is a longitudinal and cross-cultural study. It relied on children’s reports of both maternal and paternal parenting practices and styles, and multiple reporters were used to obtain the data. Moreover, unlike in most prior work, self-efficacy about both anger regulation and sadness regulation were examined. Our findings align with the differential susceptibility theory (Belsky and Pluess, 2009) and the bioecological theory of development (Bronfenbrenner and Morris, 2006), showing the need to take into account family, individual, and cultural characteristics when trying to predict youths’ wellbeing.

Indeed, as suggested by Belsky and Pluess (2009), some individuals are more affected than others by rearing experiences and environmental circumstances and individual determinants can moderate the impact of environmental factors on adolescents’ outcomes. In relation to the present study, for individuals who believe themselves to be highly capable of regulating their negative emotions, the impact of negative parenting on youths’ future adjustment is weaker than for those who do not hold this belief. Moreover, in line with the bioecological model (Bronfenbrenner and Morris, 2006), the development of self-efficacy in the domain of emotion regulation can be interpreted by considering the interactions that youths have with surrounding systems, such as family (microsystems), the larger society (exosystems), and culture (macrosystems).

This study also extends existent literature, typically targeting negative emotionality and related factors, supporting the importance of focusing on discrete negative emotions (Izard and Abe, 2003) when studying self-efficacy beliefs, their predictors, and outcomes.

This study also has some weaknesses. Multi-informant perspectives (e.g., both parent- and child-reports) were not obtained for all study variables. The samples were not nationally representative of Colombia and Italy, so caution should be used in generalizing results from the present study to those populations, as well as to populations from other countries. Even though previous studies have highlighted a high degree of comorbidity in the specific subscales within internalizing and externalizing problems (e.g., anxiety-depression and aggressive behaviors; Lilienfeld, 2003; Garnefski et al., 2005), it could be useful to examine whether the obtained results in the present study considering overall internalizing and externalizing problems as outcomes are replicated for the specific subscales.

In addition, it should be further highlighted that it was primarily self-efficacy about anger regulation that predicted change in both internalizing and externalizing problems. It would be useful for future studies to examine prediction of other developmentally important outcomes by self-efficacy beliefs about sadness regulation. Finally, it would have been useful to include culturally sensitive variables that might have clarified some of the cultural differences that emerged in the study.

## Conclusion

To our knowledge this is the first study tracing normative development of self-efficacy about both anger and sadness regulation from pre-adolescence to late adolescence, examining the association of those developmental trajectories with predictors and outcomes, in two cultural contexts, while controlling for child gender and family socio-economic status. While self-efficacy regarding anger regulation tended to increase over time, self-efficacy regarding sadness regulation did not change across age. Temperamental characteristics were predictors of the development of those self-efficacy beliefs; whereas, family characteristics were particularly useful in predicting self-efficacy regarding anger regulation. Finally, self-efficacy beliefs regarding both anger and sadness regulation were also predictive of future youth wellbeing.

## Data availability statement

The raw data supporting the conclusions of this article will be made available by the authors, without undue reservation.

## Ethics statement

The studies involving human participants were reviewed and approved by the IRB, Psychology Department, Sapienza University of Rome, Italy, and Bioethics Committee, Universidad de San Buenaventura, Medellín, Colombia. Written informed consent to participate in this study was provided by the participants’ legal guardian/next of kin.

## Author contributions

LD, CP, NE, and JL contributed to conception and design of the study. LD, JL, CP, and A-MI provided funds to support the data collection of the study. LD and CL wrote sections of the manuscript. CL performed the statistical analysis. DB, LU, EB, GG, and AF organized the database. All authors contributed to manuscript revision, read, and approved the submitted version.

## Footnotes

1 Those are unstandardized estimates. From now and beyond, all estimates reported in parentheses refer to unstandardized estimates.
