# Supplementary material for: Predictors and outcomes associated with the growth curves of self-efficacy beliefs in regard to anger and sadness regulation during adolescence: a longitudinal cross-cultural study
Source: Front Psychol. 2023 Apr 17;14:1010358. doi: 10.3389/fpsyg.2023.1010358 (PMC10149876; doi:10.3389/fpsyg.2023.1010358)
Supplement: Supplementary file 1 [file Table_1.DOCX]

Supplemental Material

Table S1

*Sample sizes differentiated by site, time, and reporter.*

|  | **Time 1** | **Time 2** | **Time 3** | **Time 4** | **Time 5** | **Time 6** |
| --- | --- | --- | --- | --- | --- | --- |
| **Colombia** | Adolescents *n=*100  Mothers *n=*101; Fathers *n=*100 | Adolescents *n=*87  Mothers *n*=83 | Adolescents *n*=84 | Adolescents  *n*=81 | Adolescents *n*=74 | Adolescents *n*=78 |
| **Italy** | Adolescents *n=*185  Mothers *n=*185; Fathers *n=*176 | Adolescents *n=*180  Mothers *n*=181 | Adolescents *n*=185 | Adolescents *n*=179 | Adolescents *n*=176 | Adolescents *n*=176 |

Table S2

*Time 1 Sociodemographic Variables*

|  | **Colombia** | **Italy** |
| --- | --- | --- |
| **Mother Years of education**  **(mean; SD)** | 11.97; 4.48 | 10.26; 5.61 |
| **Father Years of education**  **(mean; SD)** | 12.14; 4.29 | 10.01; 5.06 |

Table S3

*Descriptive statistics for study variables within the total sample.*

| **Time Variable (Informant)** | **Mean** | **SD** | **Skewness** | **Kurtosis** |
| --- | --- | --- | --- | --- |
| T1 Parents’ educational level (mother-father) | 11.34 | 4.57 | .68 | 1.72 |
| T1 Parental warmth (youth) | 3.67 | .38 | -1.38 | 1.39 |
| T1 Harsh parenting (youth) | 1.59 | .53 | 1.97 | 4.38 |
| T1 Adolescents’ internalizing problems (youth) | .39 | .23 | .58 | -.29 |
| T1 Adolescents’ externalizing problems (youth) | .28 | .17 | 1.04 | 1.18 |
| T2 Adolescents’ anger (mother) | 2.71 | .93 | .35 | -.68 |
| T2 Adolescents’ sadness (mother) | 2.27 | .72 | .74 | .17 |
| T2 Seff_Anger (youth) | 3.18 | .84 | -.25 | .19 |
| T2 Seff_Sadness (youth) | 3.20 | .77 | -.00 | .11 |
| T3 Seff_Anger (youth) | 3.19 | .89 | -.11 | -.51 |
| T3 Seff_Sadness (youth) | 3.39 | .80 | .29 | -.35 |
| T4 Seff_Anger (youth) | 2.96 | .92 | .10 | -.29 |
| T4 Seff_Sadness (youth) | 3.24 | .85 | .02 | -.39 |
| T5 Seff_Anger (youth) | 2.94 | .91 | .24 | -.32 |
| T5 Seff_Sadness (youth) | 3.32 | .84 | -.39 | -.06 |
| T6 Seff_Anger (youth) | 3.07 | .92 | .11 | -.31 |
| T6 Seff_Sadness (youth) | 3.45 | .83 | .15 | -.41 |
| T6 Adolescents’ internalizing problems (youth) | .68 | .37 | .33 | -.70 |
| T6 Adolescents’ externalizing problems (youth) | .42 | .27 | 1.09 | 1.96 |

Note: Seff_Anger = Adolescents’ self-efficacy regarding anger regulation Seff_Sadness = Adolescents’ self-efficacy regarding sadness regulation.

Table S4

*Correlations among study variables for the total sample – Anger-related variables (observed scores).*

| Variable | (1) | (2) | (3) | (4) | (5) | (6) | (7) | (8) | (9) | (10) | (11) | (12) | (13) | (14) |
| --- | --- | --- | --- | --- | --- | --- | --- | --- | --- | --- | --- | --- | --- | --- |
| (1) Adolescent gender | - |  |  |  |  |  |  |  |  |  |  |  |  |  |
| (2) Parents’ educational level | .02 | - |  |  |  |  |  |  |  |  |  |  |  |  |
| (3) T1 Parental warmth | .06 | .00 | - |  |  |  |  |  |  |  |  |  |  |  |
| (4) T1 Harsh parenting | -.05 | .05 | -.17** | - |  |  |  |  |  |  |  |  |  |  |
| (5) T1 Adolescents’ internalizing problems | .13* | -.04 | -.24** | .26** | - |  |  |  |  |  |  |  |  |  |
| (6) T1 Adolescents’ externalizing problems | -.04 | .02 | -.24** | .38** | .46** | - |  |  |  |  |  |  |  |  |
| (7) T2 Adolescents’ anger | .02 | -.41** | .07 | .04 | .08 | .04 | - |  |  |  |  |  |  |  |
| (8) T2 Seff_Anger | .03 | .05 | .17** | -.19** | -.12 | -.20** | -.17** | - |  |  |  |  |  |  |
| (9) T3 Seff_Anger | -.11 | .05 | .10 | -.17** | -.16* | -.25** | -.16** | .37** | - |  |  |  |  |  |
| (10) T4 Seff_Anger | -.06 | .09 | .06 | -.17** | -.02 | -.11 | -.18** | .24** | .41** | - |  |  |  |  |
| (11) T5 Seff_Anger | -.08 | .16* | .07 | -.09 | -.15* | -.24** | -.23** | .22** | .38** | .47** | - |  |  |  |
| (12) T6 Seff_Anger | -.07 | .15* | .02 | -.09 | -.19** | -.20** | -.37** | .24** | .37** | .41** | .54** | - |  |  |
| (13) T6 Adolescents’ internalizing problems | .28** | .13* | -.11 | .09 | .27** | .15* | .07 | -.20** | -.21** | -.16** | -.21** | -.29** | - |  |
| (14) T6 Adolescents’ externalizing problems | .06 | -.01 | .01 | .22** | .19** | .35** | .19** | -.16** | -.17** | -.26** | -.34** | -.45** | .44** | - |

*Note*: Adolescent gender was coded 0=boys, 1=girls. Seff_Anger = Adolescents’ self-efficacy regarding anger regulation. ** *p* < .01; * *p* < .05.

Table S5

*Correlations among study variables for the total sample – Sadness-related variables (observed scores).*

| Variable | (1) | (2) | (3) | (4) | (5) | (6) | (7) | (8) | (9) | (10) | (11) | (12) | (13) | (14) |
| --- | --- | --- | --- | --- | --- | --- | --- | --- | --- | --- | --- | --- | --- | --- |
| (1) Adolescent gender | - |  |  |  |  |  |  |  |  |  |  |  |  |  |
| (2) Parents’ educational level | .02 | - |  |  |  |  |  |  |  |  |  |  |  |  |
| (3) T1 Parental warmth | .06 | .00 | - |  |  |  |  |  |  |  |  |  |  |  |
| (4) T1 Harsh parenting | -.05 | .05 | -.17** | - |  |  |  |  |  |  |  |  |  |  |
| (5) T1 Adolescents’ internalizing problems | .13* | -.04 | -.24** | .26** | - |  |  |  |  |  |  |  |  |  |
| (6) T1 Adolescents’ externalizing problems | -.04 | .02 | -.24** | .38** | .46** | - |  |  |  |  |  |  |  |  |
| (7) T2 Adolescents’ sadness | .00 | -.26** | .02 | .04 | .07 | -.01 | - |  |  |  |  |  |  |  |
| (8) T2 Seff_Sadness | .08 | .15* | .09 | -.11 | -.17** | -.06 | -.17** | - |  |  |  |  |  |  |
| (9) T3 Seff_Sadness | -.18** | .07 | .11 | -.12* | -.24** | -.12* | -.19** | .30** | - |  |  |  |  |  |
| (10) T4 Seff_Sadness | -.28** | .08 | .13* | .01 | -.14** | -.11 | -.13** | .20** | .39** | - |  |  |  |  |
| (11) T5 Seff_Sadness | -.15** | .14* | .21** | .06 | -.18** | -.10 | -.16* | .23** | .43** | .50** | - |  |  |  |
| (12) T6 Seff_Sadness | -.22** | .02 | .09 | -.03 | .27** | -.11 | -.13* | .21** | .35** | .33** | .43** | - |  |  |
| (13) T6 Adolescents’ internalizing problems | .28** | .13* | -.11 | .09 | .19** | .15* | .10 | -.09 | -.25** | -.24** | -.24** | -.43** | - |  |
| (14) T6 Adolescents’ externalizing problems | .06 | -.01 | .01 | .22** | -.17** | .35** | .11 | .01 | -.03 | -.03 | .02 | -.09 | .44** | - |

*Note*: Adolescent gender was coded 0=boys, 1=girls. Seff_Sadness = Adolescents’ self-efficacy regarding sadness regulation. ** *p* < .01; * *p* < .05.

Table S6

*Descriptive statistics for study variables reported separately by cultural group.*

|  | **Colombia** | | **Italy** | |
| --- | --- | --- | --- | --- |
| **Time Variable (Informant)** | **Mean** | **SD** | **Mean** | **SD** |
| T1 Parents’ educational level (mother-father) | 10.09 | 5.05 | 12.01 | 4.14 |
| T1 Parental warmth (youth) | 3.75 | .32 | 3.62 | .39 |
| T1 Harsh parenting (youth) | 1.50 | .41 | 1.64 | .58 |
| T1 Adolescents’ internalizing problems (youth) | .36 | .22 | .40 | .23 |
| T1 Adolescents’ externalizing problems (youth) | .24 | .16 | .30 | .17 |
| T2 Adolescents’ anger (mother) | 2.77 | 1.09 | 2.68 | .84 |
| T2 Adolescents’ sadness (mother) | 2.27 | .79 | 2.26 | .68 |
| T2 Seff_Anger (youth) | 3.41 | .85 | 3.07 | .81 |
| T2 Seff_Sadness (youth) | 3.20 | .87 | 3.19 | .71 |
| T3 Seff_Anger (youth) | 3.34 | .82 | 3.11 | .91 |
| T3 Seff_Sadness (youth) | 3.34 | .82 | 3.41 | .79 |
| T4 Seff_Anger (youth) | 3.11 | 1.03 | 2.89 | .86 |
| T4 Seff_Sadness (youth) | 3.34 | .87 | 3.19 | .84 |
| T5 Seff_Anger (youth) | 3.13 | 1.06 | 2.85 | .82 |
| T5 Seff_Sadness (youth) | 3.26 | .80 | 3.35 | .84 |
| T6 Seff_Anger (youth) | 3.29 | 1.02 | 2.96 | .84 |
| T6 Seff_Sadness (youth) | 3.53 | .83 | 3.41 | .82 |
| T6 Adolescents’ internalizing problems (youth) | .60 | .34 | .71 | .37 |
| T6 Adolescents’ externalizing problems (youth) | .43 | .31 | .41 | .24 |

Note: Seff_Anger = Adolescents’ self-efficacy regarding anger regulation Seff_Sadness = Adolescents’ self-efficacy regarding sadness regulation.

Table S7

*Correlations among study variables Colombia – Anger-related variables (observed scores).*

| Variable | (1) | (2) | (3) | (4) | (5) | (6) | (7) | (8) | (9) | (10) | (11) | (12) | (13) | (14) |
| --- | --- | --- | --- | --- | --- | --- | --- | --- | --- | --- | --- | --- | --- | --- |
| (1) Adolescent gender | - |  |  |  |  |  |  |  |  |  |  |  |  |  |
| (2) Parents’ educational level | .09 | - |  |  |  |  |  |  |  |  |  |  |  |  |
| (3) T1 Parental warmth | .08 | .07 | - |  |  |  |  |  |  |  |  |  |  |  |
| (4) T1 Harsh parenting | .14 | -.04 | -.29** | - |  |  |  |  |  |  |  |  |  |  |
| (5) T1 Adolescents’ internalizing problems | .19 | -.10 | -.11 | .35** | - |  |  |  |  |  |  |  |  |  |
| (6) T1 Adolescents’ externalizing problems | .07 | .07 | -.23** | .43** | .27** | - |  |  |  |  |  |  |  |  |
| (7) T2 Adolescents’ anger | .14 | -.55** | -.00 | .13 | .04 | .08 | - |  |  |  |  |  |  |  |
| (8) T2 Seff_Anger | -.05 | .18 | .27* | -.32** | -.19 | -.22* | -.21* | - |  |  |  |  |  |  |
| (9) T3 Seff_Anger | -.25* | .13 | .11 | -.24* | -.03 | -.18 | -.19 | .31** | - |  |  |  |  |  |
| (10) T4 Seff_Anger | -.16 | .37** | -.08 | -.13 | .06 | -.02 | -.39** | .12 | .44** | - |  |  |  |  |
| (11) T5 Seff_Anger | -.18 | .44** | .17 | -.18 | -.19 | -.12 | -.44** | .24** | .26** | .48** | - |  |  |  |
| (12) T6 Seff_Anger | -.25* | .06 | -.07 | -.16 | -.19 | -.03 | -.56** | .22 | .24** | .43** | .63** | - |  |  |
| (13) T6 Adolescents’ internalizing problems | .32** | -.14 | .10 | .23* | .15 | .10 | .25* | -.08 | -.36 | -.22 | -.27* | -.38** | - |  |
| (14) T6 Adolescents’ externalizing problems | .25* | -.01 | .07 | .43** | .24* | .44** | .40** | -.14 | -.13 | -.27* | -.36** | -.48** | .51** | - |

*Note*: Adolescent gender was coded 0=boys, 1=girls. Seff_Anger = Adolescents’ self-efficacy regarding anger regulation. ** *p* < .01; * *p* < .05.

Table S8

*Correlations among study variables Italy – Anger-related variables (observed scores).*

| Variable | (1) | (2) | (3) | (4) | (5) | (6) | (7) | (8) | (9) | (10) | (11) | (12) | (13) | (14) |
| --- | --- | --- | --- | --- | --- | --- | --- | --- | --- | --- | --- | --- | --- | --- |
| (1) Adolescent gender | - |  |  |  |  |  |  |  |  |  |  |  |  |  |
| (2) Parents’ educational level | -.01 | - |  |  |  |  |  |  |  |  |  |  |  |  |
| (3) T1 Parental warmth | .04 | .02 | - |  |  |  |  |  |  |  |  |  |  |  |
| (4) T1 Harsh parenting | -.12 | .06 | -.11 | - |  |  |  |  |  |  |  |  |  |  |
| (5) T1 Adolescents’ internalizing problems | .11 | -.04 | -.27** | .22** | - |  |  |  |  |  |  |  |  |  |
| (6) T1 Adolescents’ externalizing problems | -.08 | .06 | -.22** | .34** | .54** | - |  |  |  |  |  |  |  |  |
| (7) T2 Adolescents’ anger | -.05 | -.31** | .11 | .01 | .11 | .03 | - |  |  |  |  |  |  |  |
| (8) T2 Seff_Anger | .07 | .05 | .09 | -.11 | -.07 | -.15 | -.16** | - |  |  |  |  |  |  |
| (9) T3 Seff_Anger | -.05 | .05 | .08 | -.14 | -.20** | -.27** | -.17** | .38** | - |  |  |  |  |  |
| (10) T4 Seff_Anger | .00 | -.04 | .10 | -.18* | -.05 | -.14 | -.05 | .28** | .39** | - |  |  |  |  |
| (11) T5 Seff_Anger | -.03 | .03 | .00 | -.03 | -.13 | -.28** | -.10 | .18* | .42** | .45** | - |  |  |  |
| (12) T6 Seff_Anger | .03 | .09 | .02 | -.04 | -.19* | -.25** | -.26** | .22** | .40** | .38** | .46** | - |  |  |
| (13) T6 Adolescents’ internalizing problems | .27** | .12 | -.16* | .03 | .31** | .15 | -.00 | -.22** | -.13 | -.11 | -.16* | -.22** | - |  |
| (14) T6 Adolescents’ externalizing problems | -.05 | .11 | -.03 | .22** | .19** | .34** | .04 | -.20** | -.21** | -.27** | -.34** | -.45** | .43** | - |

*Note*: Adolescent gender was coded 0=boys, 1=girls. Seff_Anger = Adolescents’ self-efficacy regarding anger regulation. ** *p* < .01; * *p* < .05.

Table S9

*Correlations among study variables Colombia – Sadness-related variables (observed scores).*

| Variable | (1) | (2) | (3) | (4) | (5) | (6) | (7) | (8) | (9) | (10) | (11) | (12) | (13) | (14) |
| --- | --- | --- | --- | --- | --- | --- | --- | --- | --- | --- | --- | --- | --- | --- |
| (1) Adolescent gender | - |  |  |  |  |  |  |  |  |  |  |  |  |  |
| (2) Parents’ educational level | .09 | - |  |  |  |  |  |  |  |  |  |  |  |  |
| (3) T1 Parental warmth | .07 | .07 | - |  |  |  |  |  |  |  |  |  |  |  |
| (4) T1 Harsh parenting | .14 | -.04 | -.29** | - |  |  |  |  |  |  |  |  |  |  |
| (5) T1 Adolescents’ internalizing problems | .19 | -.10 | -.11 | .35** | - |  |  |  |  |  |  |  |  |  |
| (6) T1 Adolescents’ externalizing problems | .07 | .07 | -.23** | .43** | .27** | - |  |  |  |  |  |  |  |  |
| (7) T2 Adolescents’ sadness | .00 | -.46** | .04 | -.03 | -.02 | -.08 | - |  |  |  |  |  |  |  |
| (8) T2 Seff_Sadness | .16 | .29* | .14 | -.16 | -.10 | -.03 | -.13 | - |  |  |  |  |  |  |
| (9) T3 Seff_Sadness | -.11 | .17 | .10 | -.07 | -.02 | -.01 | -.28** | .30** | - |  |  |  |  |  |
| (10) T4 Seff_Sadness | -.11 | .25* | .12 | .14 | .05 | -.06 | -.16 | .18 | .34** | - |  |  |  |  |
| (11) T5 Seff_Sadness | -.13 | .37** | .18 | .11 | .08 | .09 | -.25* | .18 | .40** | .38** | - |  |  |  |
| (12) T6 Seff_Sadness | -.17 | .24* | -.13 | .01 | .11 | .07 | -.36** | .19 | .38** | .28** | .45** | - |  |  |
| (13) T6 Adolescents’ internalizing problems | .32** | .06 | .09 | .23* | .15 | .10 | .21 | -.05 | -.20 | .06 | .14 | -.25* | - |  |
| (14) T6 Adolescents’ externalizing problems | .25* | -.14 | .07 | .43** | .24* | .44** | .17 | .08 | .00 | .02 | .30* | -.01 | .51** | - |

*Note*: Adolescent gender was coded 0=boys, 1=girls. Seff_Sadness = Adolescents’ self-efficacy regarding sadness regulation. ** *p* < .01; * *p* < .05.

Table S10

*Correlations among study variables Italy – Sadness-related variables (observed scores).*

| Variable | (1) | (2) | (3) | (4) | (5) | (6) | (7) | (8) | (9) | (10) | (11) | (12) | (13) | (14) |
| --- | --- | --- | --- | --- | --- | --- | --- | --- | --- | --- | --- | --- | --- | --- |
| (1) Adolescent gender | - |  |  |  |  |  |  |  |  |  |  |  |  |  |
| (2) Parents’ educational level | -.01 | - |  |  |  |  |  |  |  |  |  |  |  |  |
| (3) T1 Parental warmth | .04 | .02 | - |  |  |  |  |  |  |  |  |  |  |  |
| (4) T1 Harsh parenting | -.12 | .06 | -.11 | - |  |  |  |  |  |  |  |  |  |  |
| (5) T1 Adolescents’ internalizing problems | .11 | -.04 | -.28** | .22** | - |  |  |  |  |  |  |  |  |  |
| (6) T1 Adolescents’ externalizing problems | -.08 | -.06 | -.22** | .34** | .54** | - |  |  |  |  |  |  |  |  |
| (7) T2 Adolescents’ sadness | .00 | -.13 | .01 | .07 | .12 | .03 | - |  |  |  |  |  |  |  |
| (8) T2 Seff_Sadness | .03 | .07 | .06 | -.09 | -.20** | -.07 | -.20** | - |  |  |  |  |  |  |
| (9) T3 Seff_Sadness | -.21** | .00 | .12 | -.15* | -.23** | -.19* | -.15* | .30** | - |  |  |  |  |  |
| (10) T4 Seff_Sadness | -.35** | .02 | .12 | -.01 | -.22** | -.11 | -.11 | .21** | .41** | - |  |  |  |  |
| (11) T5 Seff_Sadness | -.16* | .01 | .23** | .04 | -.28** | -.11 | -.12 | .26** | .44** | .55** | - |  |  |  |
| (12) T6 Seff_Sadness | -.24** | -.08 | .18* | -.03 | -.26* | -.17* | -.01 | .24** | .34** | .35** | .43** | - |  |  |
| (13) T6 Adolescents’ internalizing problems | .27** | .12 | -.16* | .03 | .31* | .15 | .06 | -.12 | -.29** | -.36** | -.40** | -.51** | - |  |
| (14) T6 Adolescents’ externalizing problems | -.05 | .11 | -.03 | .15* | .17* | .34** | .07 | -.04 | -.04 | -.07 | -.14 | -.15 | .43** | - |

*Note*: Adolescent gender was coded 0=boys, 1=girls. Seff_Sadness = Adolescents’ self-efficacy in sadness regulation. ** *p* < .01; * *p* < .05.

# Table S11

# *Covariates and within T1 relations across predictors from the conditional multiple group latent growth curve model for Colombia – Model 1 Anger-related variables*

|  | Estimated | S.E. | *p* value |
| --- | --- | --- | --- |
|  |  |  |  |
| Youth gender 🡪 Intercept | -.09 | .08 | .229 |
| Youth gender 🡪 Slope | -.01 | .02 | .740 |
| Youth gender 🡪 T1 Parental warmth | .00 | -- | -- |
| Youth gender 🡪 T1 Harsh parenting | .00 | -- | -- |
| Youth gender 🡪 T1 Internalizing Problems | .07 | .02 | <.01 |
| Youth gender 🡪 T1 Externalizing Problems | .00 | -- | -- |
| Youth gender 🡪 T2 Anger | .00 | -- | -- |
| Youth gender 🡪 Parental warmth* anger | .00 | -- | -- |
| Youth gender 🡪 Harsh parenting* anger | .00 | -- | -- |
| Youth gender 🡪 T6 Internalizing problems | .17 | .04 | <.001 |
| Youth gender 🡪 T6 Externalizing problems | -.01 | .03 | .785 |
| Parents education 🡪 Intercept | **.03** | **.01** | **<.05** |
| Parents education 🡪 Slope | .000 | .002 | .926 |
| Parents education 🡪 T1 Parental warmth | .00 | -- | -- |
| Parents education 🡪 T1 Harsh parenting | .00 | -- | -- |
| Parents education 🡪 T1 Internalizing Problems | .00 | -- | -- |
| Parents education 🡪 T1 Externalizing Problems | .00 | -- | -- |
| Parents education 🡪 T2 Anger | **-.12** | **.02** | **<.001** |
| Parents education 🡪 Parental warmth* anger | -.21 | .02 | <.001 |
| Parents education 🡪 Harsh parenting* anger | .01 | .004 | <.01 |
| Parents education 🡪 T6 Internalizing problems | -.02 | .01 | <.01 |
| Parents education 🡪 T6 Externalizing problems | .01 | .003 | .095 |
| T1 Parental warmth ↔ T1 Harsh parenting | -.04 | .01 | <.001 |
| T1 Parental warmth ↔ T1 Internalizing problems | -.02 | .01 | <.001 |
| T1 Parental warmth ↔ T1 Externalizing problems | -.02 | .004 | <.001 |
| T1 Parental warmth ↔ T2 Anger | .00 | -- | -- |
| T1 Parental warmth ↔ Parental warmth* anger | **-.02** | **.01** | **<.01** |
| T1 Parental warmth ↔ Harsh parenting* anger | .00 | -- | -- |
| T1 Harsh parenting ↔ T1 Internalizing problems | .04 | .01 | <.001 |
| T1 Harsh parenting ↔ T1 Externalizing problems | .04 | .01 | <.001 |
| T1 Harsh parenting ↔ T2 Anger | .00 | -- | -- |
| T1 Harsh parenting ↔ Parental warmth* anger | .00 | -- | -- |
| T1 Harsh parenting ↔ Harsh parenting* anger | **.11** | **.02** | **<.001** |
| T1 Internalizing problems ↔ T1 Externalizing problems | .02 | .003 | <.001 |
| T1 Internalizing problems ↔ T2 Anger | .00 | -- | -- |
| T1 Internalizing problems ↔ Parental warmth* anger | .02 | .004 | <.001 |
| T1 Internalizing problems ↔ Harsh parenting* anger | **.03** | **.01** | **<.05** |
| T1 Externalizing problems ↔ T2 Anger | .00 | -- | -- |
| T1 Externalizing problems ↔ Parental warmth* anger | .004 | .003 | .186 |
| T1 Externalzing problems ↔ Harsh parenting* anger | **.03** | **.01** | **<.001** |
| T2 Anger ↔ Parental warmth* anger | **-.01** | **.03** | **.770** |
| T2 Anger ↔ Harsh parenting* anger | .08 | .22 | <.01 |
| Parental warmth* Sadness ↔ Harsh parenting* Sadness | -.02 | .01 | <.05 |
| T6 Internalizing problems ↔ T6 Externalizing problems | .02 | .01 | <.001 |

*Note.* Unstandardized betas (->) with Standard Errors and correlation coefficients (↔) are reported. Bold indicate parameters varying across cultures. -- refers to those non-significant parameters that were constrained to be equal to zero to guarantee the model parsimony.

Table S12

*Covariates and within T1 relations across predictors from the conditional multiple group latent growth curve model for Italy – Model 1 Anger-related variables*

|  | Estimated | S.E. | *p* value |
| --- | --- | --- | --- |
|  |  |  |  |
| Youth gender 🡪 Intercept | -.09 | .08 | .229 |
| Youth gender 🡪 Slope | -.01 | .02 | .740 |
| Youth gender 🡪 T1 Parental warmth | .00 | -- | -- |
| Youth gender 🡪 T1 Harsh parenting | .00 | -- | -- |
| Youth gender 🡪 T1 Internalizing Problems | .07 | .02 | <.01 |
| Youth gender 🡪 T1 Externalizing Problems | .00 | -- | -- |
| Youth gender 🡪 T2 Anger | .00 | -- | -- |
| Youth gender 🡪 Parental warmth* anger | .00 | -- | -- |
| Youth gender 🡪 Harsh parenting* anger | .00 | -- | -- |
| Youth gender 🡪 T6 Internalizing problems | .17 | .04 | <.001 |
| Youth gender 🡪 T6 Externalizing problems | -.01 | .03 | .785 |
| Parents education 🡪 Intercept | **-.003** | **.01** | **.790** |
| Parents education 🡪 Slope | .000 | .002 | .926 |
| Parents education 🡪 T1 Parental warmth | .00 | -- | -- |
| Parents education 🡪 T1 Harsh parenting | .00 | -- | -- |
| Parents education 🡪 T1 Internalizing Problems | .00 | -- | -- |
| Parents education 🡪 T1 Externalizing Problems | .00 | -- | -- |
| Parents education 🡪 T2 Anger | **-.06** | **.01** | **<.001** |
| Parents education 🡪 Parental warmth* anger | -.21 | .02 | <.001 |
| Parents education 🡪 Harsh parenting* anger | .01 | .004 | <.01 |
| Parents education 🡪 T6 Internalizing problems | -.02 | .01 | <.01 |
| Parents education 🡪 T6 Externalizing problems | .01 | .003 | .095 |
| T1 Parental warmth ↔ T1 Harsh parenting | -.04 | .01 | <.001 |
| T1 Parental warmth ↔ T1 Internalizing problems | -.02 | .01 | <.001 |
| T1 Parental warmth ↔ T1 Externalizing problems | -.02 | .004 | <.001 |
| T1 Parental warmth ↔ T2 Anger | .00 | -- | -- |
| T1 Parental warmth ↔ Parental warmth* anger | **-.02** | **.01** | **<.01** |
| T1 Parental warmth ↔ Harsh parenting* anger | .00 | -- | -- |
| T1 Harsh parenting ↔ T1 Internalizing problems | .04 | .01 | <.001 |
| T1 Harsh parenting ↔ T1 Externalizing problems | .04 | .01 | <.001 |
| T1 Harsh parenting ↔ T2 Anger | .00 | -- | -- |
| T1 Harsh parenting ↔ Parental warmth* anger | .00 | -- | -- |
| T1 Harsh parenting ↔ Harsh parenting* anger | **-.02** | **.02** | **.272** |
| T1 Internalizing problems ↔ T1 Externalizing problems | .02 | .003 | <.001 |
| T1 Internalizing problems ↔ T2 Anger | .00 | -- | -- |
| T1 Internalizing problems ↔ Parental warmth* anger | .02 | .004 | <.001 |
| T1 Internalizing problems ↔ Harsh parenting* anger | **-.01** | **.01** | **.413** |
| T1 Externalizing problems ↔ T2 Anger | .00 | -- | -- |
| T1 Externalizing problems ↔ Parental warmth* anger | .004 | .003 | .186 |
| T1 Externalzing problems ↔ Harsh parenting* anger | **-.01** | **.01** | **.052** |
| T2 Anger ↔ Parental warmth* anger | **.10** | **.02** | **<.001** |
| T2 Anger ↔ Harsh parenting* anger | .08 | .22 | <.01 |
| Parental warmth* Sadness ↔ Harsh parenting* Sadness | -.02 | .01 | <.05 |
| T6 Internalizing problems ↔ T6 Externalizing problems | .02 | .01 | <.001 |

*Note.* Unstandardized betas (->) with Standard Errors and correlation coefficients (↔) are reported. Bold indicate parameters varying across cultures. -- refers to those non-significant parameters that were constrained to be equal to zero to guarantee the model parsimony.

Table S13

*Covariates and within T1 relations across predictors from the conditional multiple group latent growth curve model for Colombia – Model 2 Sadness-related variables*

|  | Estimated | S.E. | *p* value |
| --- | --- | --- | --- |
|  |  |  |  |
| Youth gender 🡪 Intercept | .11 | .08 | .145 |
| Youth gender 🡪 Slope | -.05 | .02 | <.01 |
| Youth gender 🡪 T1 Parental warmth | .00 | -- | -- |
| Youth gender 🡪 T1 Harsh parenting | .00 | -- | -- |
| Youth gender 🡪 T1 Internalizing Problems | .07 | .02 | <.01 |
| Youth gender 🡪 T1 Externalizing Problems | .00 | -- | -- |
| Youth gender 🡪 T2 Sadness | .00 | -- | -- |
| Youth gender 🡪 Parental warmth* Sadness | .00 | -- | -- |
| Youth gender 🡪 Harsh parenting* Sadness | .00 | -- | -- |
| Youth gender 🡪 T6 Internalizing problems | .03 | .09 | .752 |
| Youth gender 🡪 T6 Externalizing problems | .003 | .05 | .954 |
| Parents education 🡪 Intercept | .02 | .01 | .078 |
| Parents education 🡪 Slope | -.003 | .002 | .243 |
| Parents education 🡪 T1 Parental warmth | .00 | -- | -- |
| Parents education 🡪 T1 Harsh parenting | .00 | -- | -- |
| Parents education 🡪 T1 Internalizing Problems | .00 | -- | -- |
| Parents education 🡪 T1 Externalizing Problems | .00 | -- | -- |
| Parents education 🡪 T2 Sadness | **-.07** | **.02** | **<.001** |
| Parents education 🡪 Parental warmth* Sadness | .01 | .003 | <.01 |
| Parents education 🡪 Harsh parenting* Sadness | .00 | -- | -- |
| Parents education 🡪 T6 Internalizing problems | .01 | .01 | .302 |
| Parents education 🡪 T6 Externalizing problems | **-.01** | **.01** | **.075** |
| T1 Parental warmth ↔ T1 Harsh parenting | -.04 | .01 | <.01 |
| T1 Parental warmth ↔ T1 Internalizing problems | -.02 | .01 | <.001 |
| T1 Parental warmth ↔ T1 Externalizing problems | -.01 | .004 | <.001 |
| T1 Parental warmth ↔ T2 Sadness | .00 | -- | -- |
| T1 Parental warmth ↔ Parental warmth* Sadness | **.02** | **.01** | **<.01** |
| T1 Parental warmth ↔ Harsh parenting* Sadness | **-.001** | **.008** | **.886** |
| T1 Harsh parenting ↔ T1 Internalizing problems | .03 | .01 | <.001 |
| T1 Harsh parenting ↔ T1 Externalizing problems | .03 | .01 | <.001 |
| T1 Harsh parenting ↔ T2 Sadness | -.002 | .01 | .799 |
| T1 Harsh parenting ↔ Parental warmth* Sadness | **.00** | **--** | **--** |
| T1 Harsh parenting ↔ Harsh parenting* Sadness | **.02** | **.01** | **<.05** |
| T1 Internalizing problems ↔ T1 Externalizing problems | **.01** | **.004** | **<.01** |
| T1 Internalizing problems ↔ T2 Sadness | .00 | -- | -- |
| T1 Internalizing problems ↔ Parental warmth* Sadness | .00 | -- | -- |
| T1 Internalizing problems ↔ Harsh parenting* Sadness | .01 | .004 | <.01 |
| T1 Externalizing problems ↔ T2 Sadness | .00 | -- | -- |
| T1 Externalizing problems ↔ Parental warmth* Sadness | .00 | -- | -- |
| T1 Externalizing problems ↔ Harsh parenting* Sadness | .01 | .003 | <.001 |
| T2 Sadness ↔ Parental warmth* Sadness | **-.003** | **.02** | **.874** |
| T2 Sadness ↔ Harsh parenting* Sadness | .04 | .01 | <.001 |
| Parental warmth* Sadness ↔ Harsh parenting* Sadness | **-.03** | **.01** | **<.001** |
| T6 Internalizing problems ↔ T6 Externalizing problems | .03 | .01 | <.01 |

*Note.* Unstandardized betas (->) with Standard Errors and correlation coefficients (↔) are reported. Bold indicate parameters varying across cultures. -- refers to those non-significant parameters that were constrained to be equal to zero to guarantee the model parsimony.

Table S14

*Covariates and within T1 relations across predictors from the conditional multiple group latent growth curve model for Italy – Model 2 Sadness-related variables*

|  | Estimated | S.E. | *p* value |
| --- | --- | --- | --- |
|  |  |  |  |
| Youth gender 🡪 Intercept | .11 | .08 | .145 |
| Youth gender 🡪 Slope | -.05 | .02 | <.01 |
| Youth gender 🡪 T1 Parental warmth | .00 | -- | -- |
| Youth gender 🡪 T1 Harsh parenting | .00 | -- | -- |
| Youth gender 🡪 T1 Internalizing Problems | .07 | .02 | <.01 |
| Youth gender 🡪 T1 Externalizing Problems | .00 | -- | -- |
| Youth gender 🡪 T2 Sadness | .00 | -- | -- |
| Youth gender 🡪 Parental warmth* Sadness | .00 | -- | -- |
| Youth gender 🡪 Harsh parenting* Sadness | .00 | -- | -- |
| Youth gender 🡪 T6 Internalizing problems | .03 | .09 | .752 |
| Youth gender 🡪 T6 Externalizing problems | .003 | .05 | .954 |
| Parents education 🡪 Intercept | .02 | .01 | .078 |
| Parents education 🡪 Slope | -.003 | .002 | .243 |
| Parents education 🡪 T1 Parental warmth | .00 | -- | -- |
| Parents education 🡪 T1 Harsh parenting | .00 | -- | -- |
| Parents education 🡪 T1 Internalizing Problems | .00 | -- | -- |
| Parents education 🡪 T1 Externalizing Problems | .00 | -- | -- |
| Parents education 🡪 T2 Sadness | **-.03** | **.01** | **<.05** |
| Parents education 🡪 Parental warmth* Sadness | .01 | .003 | <.01 |
| Parents education 🡪 Harsh parenting* Sadness | .00 | -- | -- |
| Parents education 🡪 T6 Internalizing problems | .01 | .01 | .302 |
| Parents education 🡪 T6 Externalizing problems | **.004** | **.01** | **.382** |
| T1 Parental warmth ↔ T1 Harsh parenting | -.04 | .01 | <.01 |
| T1 Parental warmth ↔ T1 Internalizing problems | -.02 | .01 | <.001 |
| T1 Parental warmth ↔ T1 Externalizing problems | -.01 | .004 | <.001 |
| T1 Parental warmth ↔ T2 Sadness | .00 | -- | -- |
| T1 Parental warmth ↔ Parental warmth* Sadness | **.01** | **.01** | **.254** |
| T1 Parental warmth ↔ Harsh parenting* Sadness | **-.04** | **.01** | **<.001** |
| T1 Harsh parenting ↔ T1 Internalizing problems | .03 | .01 | <.001 |
| T1 Harsh parenting ↔ T1 Externalizing problems | .03 | .01 | <.001 |
| T1 Harsh parenting ↔ T2 Sadness | -.002 | .01 | .799 |
| T1 Harsh parenting ↔ Parental warmth* Sadness | **.00** | **--** | **--** |
| T1 Harsh parenting ↔ Harsh parenting* Sadness | **.11** | **.02** | **<.001** |
| T1 Internalizing problems ↔ T1 Externalizing problems | **.02** | **.003** | **<.001** |
| T1 Internalizing problems ↔ T2 Sadness | .00 | -- | -- |
| T1 Internalizing problems ↔ Parental warmth* Sadness | .00 | -- | -- |
| T1 Internalizing problems ↔ Harsh parenting* Sadness | .01 | .004 | <.01 |
| T1 Externalizing problems ↔ T2 Sadness | .00 | -- | -- |
| T1 Externalizing problems ↔ Parental warmth* Sadness | .00 | -- | -- |
| T1 Externalizing problems ↔ Harsh parenting* Sadness | .01 | .003 | <.001 |
| T2 Sadness ↔ Parental warmth* Sadness | **-.08** | **.01** | **<.001** |
| T2 Sadness ↔ Harsh parenting* Sadness | .04 | .01 | <.001 |
| Parental warmth* Sadness ↔ Harsh parenting* Sadness | **-.03** | **.01** | **<.001** |
| T6 Internalizing problems ↔ T6 Externalizing problems | .03 | .01 | <.01 |

*Note.* Unstandardized betas (->) with Standard Errors and correlation coefficients (↔) are reported. Bold indicate parameters varying across cultures. -- refers to those non-significant parameters that were constrained to be equal to zero to guarantee the model parsimony.
